# Mining dynamic noteworthy functions in software execution sequences

**DOI:** 10.1371/journal.pone.0173244

**Published:** 2017-03-09

**Authors:** Bing Zhang, Guoyan Huang, Yuqian Wang, Haitao He, Jiadong Ren

**Affiliations:** 1 College of Information Science and Engineering, Yanshan University, Qinhuangdao, Hebei, China; 2 The Key Laboratory for Computer Virtual Technology and System Integration of Hebei Province, Qinhuangdao, Hebei, China; Tianjin University, CHINA

## Abstract

As the quality of crucial entities can directly affect that of software, their identification and protection become an important premise for effective software development, management, maintenance and testing, which thus contribute to improving the software quality and its attack-defending ability. Most analysis and evaluation on important entities like codes-based static structure analysis are on the destruction of the actual software running. In this paper, from the perspective of software execution process, we proposed an approach to mine dynamic noteworthy functions (*DNFM*)in software execution sequences. First, according to software decompiling and tracking stack changes, the execution traces composed of a series of function addresses were acquired. Then these traces were modeled as execution sequences and then simplified so as to get simplified sequences (*SFS*), followed by the extraction of patterns through pattern extraction (*PE*) algorithm from *SFS*. After that, evaluating indicators *inner-importance* and *inter-importance* were designed to measure the noteworthiness of functions in *DNFM* algorithm. Finally, these functions were sorted by their noteworthiness. Comparison and contrast were conducted on the experiment results from two traditional complex network-based node mining methods, namely *PageRank* and *DegreeRank*. The results show that the *DNFM* method can mine noteworthy functions in software effectively and precisely.

## Introduction

The identification of important entities (function, class, method, implement, etc.) has a remarkable theoretical and practical significance for software designing, development, maintenance and management. For instance, software reliability can be increased by special protection on influential nodes in software. Studies on these noteworthy entities can not only help cut the workload of software testing but also help enhance the accuracy of software maintenance, thus reducing the maintenance costs. Thus, to improve software maintenance and development is an involved and costly task. According to Gartner, global software expenditures for 2010 amounted to $229 billion, with large vendors such as Microsoft and IBM reporting multi-billion dollar costs for software development each year. Most of the development cost—an estimated 50 to 90 percent of the total costs—is due to software maintenance. Despite the high costs, software is notoriously unreliable, and software bugs can wreak havoc on software producers and consumers alike—a *NIST* survey estimated the annual cost of software bugs to be about $59.5 billion. Therefore, knowledge on improving the maintenance by effectively identifying which components to debug, test, or refactor motivated us to establish suitable model for software and design a method to mine dynamic noteworthy functions in software system [1].

Today, complex network is always a research hotspot [2–4]. Many researchers study the characteristics of complex network in software system [5–7]. Studies show that software systems have complex network phenomena, namely small-world effect [8] and scale-free property [9]. With further research on software systems, the development and maturing of complex network theory [10, 11] provides a series of classic ways [12–16] for software functions analysis. It is worthy to be noticed that there have been many well-known researches in this field before. *Bonacich* et al. [17] proposed an algorithm using degree centrality [18–21] to evaluate the importance of nodes for the first time in 1972, seen from which the bigger degree centrality is, the more important a node is. In addition, *Brin* and *Page* [22] presented a *PageRank* algorithm where a Web page’s rank in search results is determined by the number of other pages link to it. Subsequently, methods based on *betweenness* [23, 24], *closeness* [25], *eigenvector* [26] and other metrics appeared gradually. Almost all these methods model software structure as complex networks, suggesting that a large number of complex links among nodes make nodes noteworthy. Besides, many new algorithms also have been proposed gradually, such as *HITS* [27], *LeaderRank* [28]], *NodeRank* [29]. Such algorithms are all based on random-walk model. They take connectivity among nodes and the importance of neighborhood nodes into consideration to determine the importance of nodes. And most of them have been applied in undirected and unweighted network, which still has some certain limitations.

Mapping the software structure or execution traces to complex networks, on which the mining of crucial nodes have certain effects and advantages. However, relevant methods are only designed from a static point of view to analyze software network and to measure the importance of nodes through dependency and connectivity among nodes, such as betweenness, in-degree, out-degree [30]. What’s more, in the process of constructing the network model, these will ignore some dynamic characteristics of the software in the execution process, such as the order of functions, loop, recursion and other control forms, the length of function set. Therefore, if the software structure is analyzed only from the perspective of complex network, dynamic behavior characteristics of software execution process couldn’t be captured accurately [31].

Based on the above mentioned, we proposed a dynamic noteworthy functions mining(*DNFM*) algorithm to help evaluate functions from the perspective of dynamic behavior characteristics in software execution sequences. Firstly, we decompiled software, traced the change of stack during software execution process so as to obtain original software execution traces formed by a series of function addresses. Then, we modeled these original traces as execution sequences and simplified them by Repetitive patterns eliminating algorithm [32], and designed a pattern extraction (*PE*) algorithm to extract patterns from *SFS*. After that, we designed two metrics *inner-importance* and *inter-importance* which are aimed to measure noteworthiness of functions in *DNFM* algorithm, followed by the categorization of functions according to their noteworthiness. Finally, we did three groups of experiment, and compared the results of *DNFM* with the results of two classic algorithms *PageRank* and *DegreeRank*.

## Methods

### Relevant definitions

*DNFM* approach analyzes the characteristics of software mainly at function level. Since the execution process of software is mostly the mutual call procedure between functions, software execution traces can be modeled as function call sequences expressed as follows.

$$\begin{array}{c} S=<S_{1}\ldots S_{i}\ldots S_{n}>\left(1<i<n\right) \end{array}$$ (1)

**Fig 1:**
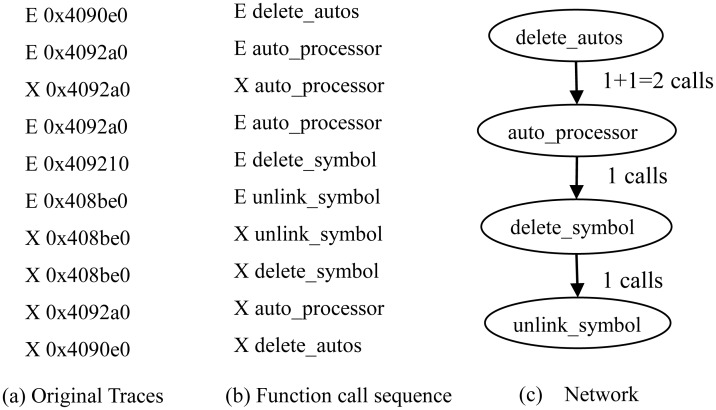
Modeling original Traces as function call sequence and network.

In *S*, *S*_*i*_ represents a feature vector including all information of a function in software execution process, which is expressed as < *Flg*, *Fname* >. Here, *Flg* is the entrance-exit flag to marked functions. As the function is a caller, *Flg* can be denoted as *E*, otherwise be *X*. *Fname* is the name of function. These processes are shown in Fig 1(a) and 1(b) in more detail.

The sequence *S* is composed of a series of continuous marked functions orderly. Thus, *S* can accurately reflect the actual running process of software, especially the order of function calling and the relationship between different functions. As shown in Fig 1(c), the original traces are modeled as complex network, the weight on edges are only equal to the times of function addresses with flag *E* appearing in traces. Seen from the network, the actual process of function calling is not clear yet.

In *S*, there exist many execution-sequence-pattern (*ESP*), which are defined as follows.

**Definition 1** ***ESP (execution-sequence-pattern)*** *ESP* for function call sequences is defined as *P*(< *Si*, …, *Sj* >) where *Si* and *Sj* are the elements derived from sequence *S*, satisfies: *Si*. *Flg* = *E*, *Si*.*FName*(*fi*) = *Sj*.*Fname*(*fj*), *Sj*.*Flg* = *X*. Besides, all flags and functions between *Si* and *Sj* are in pair, for instance, sequence segments like < *E*, *A* >, < *E*, *B* >, < *E*, *A* >, < *X*, *A* >, < *X*, *B* >, < *X*, *A* > is an *ESP*, but < *E*, *A* >, < *E*, *B* >, < *E*, *A* >, < *X*, *A* > isn’t, although it meets the condition. Besides, one function call sequence can own many different *ESP*, and each *ESP* can appear many times in *S*.

In practice, the relationship between different *ESP* is very complex. Due to loops, an execution trace may contain repeated interesting patterns. What’s more, different function call relations would result in different relationships between *ESP*. Suppose there exist *ESP*_1_ = *Sp*, …, *Sq*(*p* < *q*) and *ESP*_2_ = *Ss*, …, *St*(*s* < *t*) in *S*, there may exist three relationships between these two *ESP* which are shown below.

1. As *S* = *S*1, …, *ESP*_1_, *ESP*_2_, …, *Sn*, when *ESP*_1_ = *ESP*_2_, *ESP*_1_ and *ESP*_2_ are reciprocal **Repetitive-pattern**.
2. As *S* = *S*1, …, *ESP*_1_, …, *Sn*, when *ESP*_1_ ⊃ *ESP*_2_, *ESP*_1_ and *ESP*_2_ are reciprocal **Contain-Pattern**.
3. As *S* = *S*1, …, *ESP*_1_, *ESP*_2_, …, *Sn*, when *ESP*_1_ ≠ *ESP*_2_, *ESP*_1_ and *ESP*_2_ are reciprocal **Parallel-pattern**.

Let *I* < *f*1, *f*2, …, *fm* > be a set of functions which has appeared in *S*. When *S* is simplified by the Repetitive patterns eliminating algorithm, *SFS* is obtained. According to the definition of *Frequency* of function and *Length of* ESP, two indexes *inner-importance* and *inter-importance* for each function in set *I* can be calculated, which are used to measure the noteworthiness of functions. The related definitions are as follows.

**Definition 2** ***Frequency*** Frequency indicates how many times an *ESP* or a function occurs in the simplified function call sequence. For an *ESP*, it is denoted as *F*(*p*), while for a function, it is denoted as *F*(*f*).

**Definition 3** ***Length of ESP*** Length of *ESP* is the number of vectors in a *ESP*, such as, the length of pattern *P*(< *S*1, *S*2 …, *Sm* >), which is denoted by *L*(*P*), is *m*.

**Definition 4** ***inner-importance (IN)*** *IN* means the probability of function *fi* which appears in *SFS*. It is defined as follows.

$$\begin{array}{c} IN\left(f_{i}\right)=\frac{F(f_{i})}{L(SFS)} \end{array}$$ (2)

Here, *F*(*fi*) is the frequency of function *fi*, and *L*(*SFS*) is the length of simplified function call sequence *SFS*.

**Definition 5** ***inter-importance (IT)*** In *S*, the function *fi* may appear in many different *ESPs* such as *P*1, *P*2, *P*3…*Pm*, and each *ESP* may appear many times. Thus, the *inter-importance* of each function can be defined as below.

$$\begin{array}{c} IT\left(f_{i}\right)=\frac{\sum_{i=1}^{m}\left(\left(F\left(P_{i}\right)*L\left(P_{i}\right)\right)\right)}{L(SFS)*L(SFS)} \end{array}$$ (3)

Here, *F*(*pi*) is the frequency of *Pi*, and *L*(*pi*) is the length of *Pi*, and *L*(*pi*) is the length of *Pi*. Accordingly, *L*(*SFS*) is the length of simplified function call sequence *SFS*.

**Definition 6** ***Noteworthiness*** The noteworthiness of function *D*(*fi*) is determined by the combination of its *inner-importance* and *inter-importance*, which is defined as below.

$$\begin{array}{c} D\left(f_{i}\right)=\sqrt{{\left(IN\left(f_{i}\right)\right)}^{2}+IT{\left(f_{i})\right)}^{2}} \end{array}$$ (4)

**Definition 7** ***Shared-node rate*** *Shared-Node* rate describes the possibility that a node in set *ρ* occurs in set *σ* or *τ*. Let the number of elements in them all equate *N*, then *Shared-Node* rate equals the number of nodes shared by *ρ* and *σ* ∪ *τ* divided by *N*, which is represented as follows.

$$\begin{array}{c} \Psi \left(\rho ,\sigma ,\tau \right)=\frac{|\rho \cap (\sigma \cup \tau )|}{N} \end{array}$$ (5)

Similarly, the ***Node similarity*** can be defined as below.

$$\begin{array}{c} \Gamma \left(\rho ,\sigma \right)=\frac{|\rho \cap \sigma )|}{N} \end{array}$$ (6)

### PageRank and DegreeRank

*PageRank* algorithm was created to rank web pages based on the number of other web pages linking to it. The *PageRank* of one web page is defined as follows.

$$\begin{array}{c} PageRank\left(p_{i}\right)=\frac{1-q}{N}+q\sum_{p_{j}\in M\left(p_{i}\right)}\frac{PageRank(p_{j})}{L(p_{j})} \end{array}$$ (7)

In formula (7), *p*_1_, *p*_2_, …, *p*_*N*_ denote a series of pages, *M*(*p*_*i*_) is the set of pages which link to page *p*_*i*_, and *L*(*p*_*j*_) is the set of pages to which pape *p*_*i*_ links. *N* is the number of total pages, and *d*(0 < *d* < 1) is a decay factor. In addition, *q* denotes teleporting, which means that the user maybe skip to another random web page from the current web page with a very low probability, and there is no hyperlink between the two web pages. Considered that the user couldn’t skip from the current web page to the random web page directly, *q* is designed to describe the probability all out of pure mathematics sense.

The *DegreeRank* algorithm considers that the bigger the degree of note is, the more important the node is. Suppose there is an undirected graph which is formed of *g* nodes. The degree of node *v*_*i*_ *C*_*D*_ is the number of links between *v*_*i*_ and nearest neighborhood nodes, which is defined as below.

$$\begin{array}{c} C_{D}\left(N_{i}\right)=\sum_{j=1}^{g}x_{ij}\left(i\neq j\right) \end{array}$$ (8)

Where *C*_*D*_(*N*_*i*_) is the degree of *v*_*i*_, $\sum_{j=1}^{g}x_{ij}$ is the number of links between *v*_*i*_ and nearest neighborhood nodes except the links between *vi* and itself.

### Framework of DNFM approach

**Fig 2:**
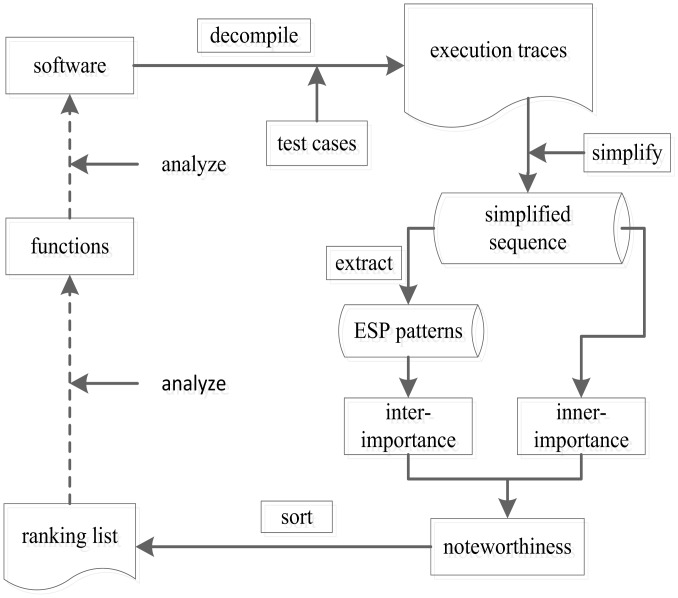
Framework of *DNFM* approach.

*DNFM* approach can capture the dynamic behavioral characteristics of software execution process effectively, and in the meanwhile it can consider the properties of nodes and patterns in a function call sequence. The whole process for *DNFM* is shown in Fig 2. At first, we decompiled the software by different test cases to obtain the software execution traces, and mapped them to function call sequences. In order to reduce the impact result from loop structures and respective patterns on *DNFM* algorithm, we applied the Repetitive patterns eliminating algorithm to simplify the function call sequence. After that, we extracted patterns from *SFS*. Accordingly, we set two metrics *inner-importance* and *inter-importance* combined to measure the noteworthiness of functions. At last, we sorted out functions by the noteworthiness and formed a function ranking list.

### Repetitive patterns eliminating algorithm

In general, the function call sequence is very complex and numerous. Due to loops, a trace can have repeated patterns. That’s to say, this means that there exist a large number of repetitive patterns, which will waste a lot of time during algorithm execution process. In order to improve the efficiency of the algorithm and make the sequence clearer, it is essential to delete continuous repeated patterns in *S*. The pseudo code of the Repetitive patterns eliminating algorithm [24] is shown as below.

**Algorithm 1: Repetitive patterns eliminating algorithm**

**Inputs**: *S*;

**Output**: the simplified function call sequence (*SFS*);

1. **for** all *ESP* in *S*

2.  **if** exists *m* Repetitive patterns *p*, *p*_1_, *p*_2_, …, *p*_*m*−1_ in *S*

3.   Delete (*p*_1_, *p*_2_, …, *p*_*m*−1_)

4.    *p* → *SFS*

5.  **end if**

6. **end for**

In Line 1, the algorithm scans the original function call sequence *S* in searching all continuous repeated patterns. In Line 2 to Line 3, if there exist continuous repeated *ESP*, the algorithm will judge whether all flag have been matched so as to ensure all functions’ entrance-flag are matched to its corresponding exit-flag, and then the repeated patterns are deleted. As shown in Line 4, an *ESP* *p* is left for *SFS*. At last, the *SFS* is generated.

**Fig 3:**
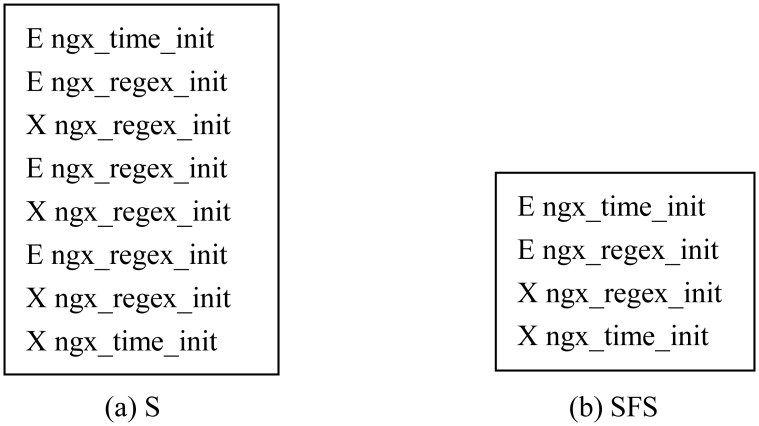
An example about simplifying *S* to *SFS*.

Suppose a function call sequence is shown as Fig 3(a), there exist obviously repetitive patterns because the *ESP* < (*E* *ngx*_*regex*_*init*), (*Xngx*_*regex*_*init*) > is executed three times continuously. Repeated *ESP* would waste a lot of time during algorithm execution process and increase noteworthiness of function *ngx*_*regex*_*init*, we need to delete those repeated *ESP*. Thus we obtain the simplified function call sequence which is shown as Fig 3(b).

### Pattern Extraction algorithm

Seen from the definitions of inner-importance and inter-importance, we know that the noteworthiness of functions is related to *ESP*, including its frequency *F*(*P*) and its length *L*(*P*). However, the elements in *SFS* are mainly function vectors like < *Flg*, *Fname* >, thus in this section, we designed a Pattern Extraction algorithm to extract all patterns from *SFS*, of which the pseudo codes are shown as follows.

**Algorithm 2: Pattern Extraction algorithm**

**Inputs**: *SFS*

**Output**: *ESP* set *P*_*set*_

1. **for** each vector *S*_*i*_ in *SFS*

2.  **if** (*S_i_*.*flag* == ‘*E*’)

3.   **for** each vectors *S*_*j*_(*i* < *j*) in *SFS*

4.    **if** (*S_j_*.*flag* == ‘*X*’ and *S*_*i*_.*Fname* = = *S*_*j*_.*Fname*)

5.     **if** (*checkEX*(*Si*, *Sj*)) //check whether flags of entrance-exit are matched

6.      *ESP* ← *P*(*S*_*i*_, *S*_*j*_)

7.     **end if**

8.     **if** (*CheckPattern*(*ESP*, *P*_*set*_)) //check whether there exist the same pattern

9.      *F*(*P*)++; //frequency of the pattern adds 1

10.     **else**

11.      add_pattern (*ESP*, *P*_*set*_); // save the pattern in *P*_*set*_

12.     **end if**

13.    **end if**

14.   **end for**

15.  **end if**

16.**end for**

Line 1 to Line 3 of algorithm 2 traverse the elements in the whole simplified function call sequence. For a vector Si in *SFS*, if its entrance-exit flag is in pairs and correspond function name *S*_*j*_. *Fname* is the same as the *Fname* of *S*_*i*_, from which it can be judged that the sequence between *S*_*i*_ and *S*_*j*_ can form an *ESP*, these processes are shown from Line 4 to Line 6. Then, in Line 8 to Line 9, as the pattern exists in *P*_*set*_, the frequency of the pattern increases one. Otherwise, the pattern *ESP* is put into *P*_*set*_ in Line 11. In the whole process, each vector in *SFS* can be checked and all patterns can be obtained to form *P*_*set*_.

### DNFM algorithm

During the above process, the simplified function call sequence *SFS* and *ESP* *set* *P*_*set*_ are obtained. After that, the *inter-importance* and *inner-importance* are computed and the *Noteworthiness* for each functions in *I* is got as well. Then, the noteworthiness of functions can be sorted by its *Noteworthiness*. Here, we propose a *DNFM* algorithm to conduct it. The pseudo-code is described as follows.

**Algorithm 3: DNFM algorithm**

**Inputs**: *SFS*; *P*_*set*_; *I*

**Output**: function rank list

1. **for** each *f*_*i*_ in *I*

2.  **for** each *S*_*i*_ in *SFS*

3.   **if** (*S*_*i*_.*Fname* = = *f*_*i*_)

4.    *F*(*f*_*i*_)++;

5.    *F*(*p*_*i*_)++;

6.   **end if**

7.  **end for**

8.  *INi* = *calculateIN*(*F*(*f*_*i*_), *L*(*SFS*));

9.  **for** each *p*_*i*_ in *P*_*set*_

10.   **if**(*f*_*i*_ *in* *p*_*i*_)

11.    *Lt*+ = *F*(*p*_*i*_)*L(*p*_*i*_)

12.   **end if**

13.  **end for**

14.  *IT*_*i*_ = *calculateIT*(*L*_*t*_, *L*(*SFS*));

15.  *D*(*f*_*i*_) = *Calculate*(*IN*_*i*_, *ITi*);//calculate the Noteworthiness

16. **end for**

17. function rank list ← Sort(Noteworthiness); //sort functions by Noteworthiness

18. **return** function rank list;

First, in order to calculate the inner-importance of each function, the algorithm analyzes each vector in *SFS* and counts the frequency of each function and its corresponding patterns, which are shown in Line 4 and Line 5. Then, the algorithm calculates the *inner-importance* for each function according to the definition 4 in Line 8. Suppose that there is a function *f*_*i*_ whose frequency is 6 according to the execution result of the algorithm and the length of *SFS* is 100, thus, the *IN*_*i*_ should be 6 divided by 100 on the basis of formula of *inner-importance*.

Then the *inter-importance* is computed. For each function *f* in set *I*, there might exist more than one *ESP* whose frequency and length are different. Assume that function *f*_*i*_ in *ESP* *p*_1_, *p*_2_, whose length and frequency are 5, 1 and 10, 2 respectively, thus *IT*_*j*_ is equal 0.0025 according to the definition 5 in Line 14. *IT* is actually an accumulative value. When the algorithm scans a new *ESP*, the amount of *IT* might be accumulated until all *ESP* have been found.

After that, the *DNFM* algorithm calculates the *Noteworthiness* for each functions by combining its *inner-importance* and *inter-importance* in Line 15. Finally, all functions can be sorted by *Noteworthiness* in line 17, thus we can obtain the top-k noteworthy functions in function rank list. It is noted that the higher a function ranks, the more noteworthy a function is.

## Results

### Objects and data

In this section, three software are used for the experiments. (1) Nginx- a lightweight web server reverse proxy server and e-mail (imappop3) proxy server using bsd-like agreement. (2) Deadbeef-a good music player which can play cue, mp3, ogg, flac, ape music file and so on. (3) Cflow- a tool to analyze a collection of *C* source files and print a graph, charting control flow within the program. We decompile the software and trace the change of stack after processing different operations on them so as to obtain some complete software execution traces from three different software. Accordingly three algorithms including *DNFM*, *PageRank* and *DegreeRank* were performed on the model established by these complete software execution traces for analyzing complex software structure and finding most noteworthy functions.

### Comparative analysis

In this part, we compare the *DNFM* algorithm with *PageRank* and *DegreeRank*, the two classic algorithms on mining important nodes based on complex network. Thus, we perform the three algorithm on the three open source software *Nginx*, *Deadbeef* and *Cflow*, and do the contrast analysis on experiment results.

The experiments are conducted on *64 bit Windows 7 ultimate*, *Xeon CPU E5-2603 @1.80GHz*, *16G* of *RAM* and *Ubuntu14.04*. The length of original data sequence (LOS) and simplified sequence (LSS), and time consumption(TC) for removing adjacent repetitive patterns in experiments are listed in Table 1.

**Table 1:** LOS, LSS and TC in each trial for different software.

	Nginx	Deadbeef	Cflow
LOS	6450	13048	7276
LSS	2680	5110	3914
TC	106.336s	461.275s	224.787s

In Table 1, the Repetitive patterns eliminating algorithm is effective to eliminate the adjacent repetitive patterns, however, it is a waste of time. As the software execution sequence length increases, execution time becomes longer. Despite of this, what we concerned about is to mine dynamic noteworthy functions efficiently.

**Fig 4:**
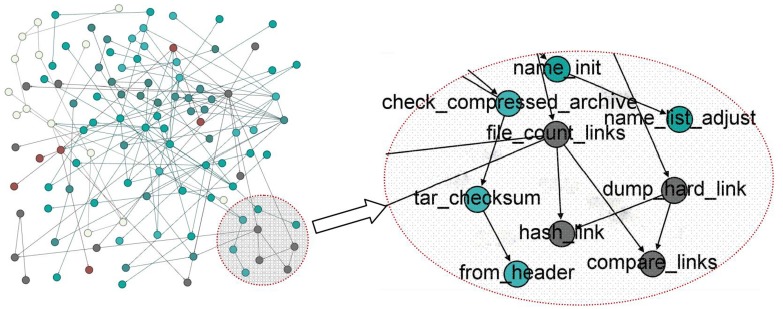
Software Complex Network.

Before performing *PageRank* and *DegreeRank* algorithm, the software execution traces need to be modeled as complex network *G* = < *V*, *E*, *W* >. If there exists an edge between node *V*_*i*_ and *V*_*j*_, the weight is assigned as 1, otherwise is 0. The networks are similar with the network displayed in Fig 4. What’s the different are the number of nodes and edges for different software networks. Here, Table 2 gives these parameters for different software complex networks.

**Table 2:** The parameters of software complex networks.

	Nginx	Deadbeef	Cflow
Nodes	244	162	100
Edges	517	436	144

Based on software complex network and function call sequence, the functions were ranked, which are shown in Tables 3, 4 and 5. Here, we treat the result of *DNFM* algorithm as bases, and list top 15 functions compared with the top 15 functions in the ranking list got by *PageRank* and *DegreeRank* algorithm. ′′−′′ means the function is not in the top 15 function list of *PageRank* or *DegreeRank*. The number represents the order of a function in top 15 function list of *DNFM*.

**Table 3:** Results of three algorithms performed on *Nginx*.

Fname	Noteworthiness	DNFM	PageRank	DegreeRank
*ngx*_*palloc*	0.185821	No.1	No.1	No.9
*ngx*_*pcalloc*	0.086567	No.2	No.2	No.2
*ngx*_*array*_*push*	0.081343	No.3	No.5	No.3
*ngx*_*pnalloc*	0.045522	No.4	No.10	-
*ngx*_*http*_*merge*_*locations*	0.032836	No.5	-	No.7
*ngx*_*array*_*init*	0.029104	No.6	No.8	No.10
*ngx*_*strlow*	0.026119	No.7	-	-
*ngx*_*hash*_*key*	0.022388	No.8	-	-
*ngx*_*conf*_*read*_*token*	0.021642	No.9	-	-
*ngx*_*http*_*merge*_*servers*	0.020896	No.10	-	No.8
*ngx*_*hash*_*add*_*key*	0.020149	No.11	-	-
*ngx*_*alloc*	0.017164	No.12	No.7	-
*ngx*_*conf*_*handler*	0.013437	No.13	-	No.11
*ngx*_*cpystrn*	0.013433	No.14	No.13	-
*ngx*_*http*_*add*_*variable*	0.010448	No.15	No.9	No.14
Node similarity (Γ)			8/15	8/15
Shared-Node Rate (Ψ)			10/15	

**Table 4:** Results of three algorithms performed on *Deadbeef*.

Fname	Noteworthiness	DNFM	PageRank	DegreeRank
*mutex*_*lock*	0.168689	No.1	No.2	No.6
*mutex*_*unlock*	0.167906	No.2	No.1	No.10
*pl*_*lock*	0.081409	No.3	No.3	No.4
*pl*_*unlock*	0.081409	No.4	No.4	No.3
*conf*_*unlock*	0.060274	No.5	No.5	No.12
*conf*_*lock*	0.060274	No.6	No.6	No.8
*conf*_*get*_*str*_*fast*	0.045010	No.7	No.9	No.15
*conf*_*get*_*int*	0.025832	No.8	-	No.7
*plt*_*unref*	0.015656	No.9	-	-
*plt*_*ref*	0.015656	No.10	-	-
*plug*_*get*_*output*	0.014873	No.11	-	-
*tf*_*compile*_*plain*	0.013699	No.12	No.12	-
*conf*_*get*_*str*	0.009393	No.13	-	No.13
*conf*_*set*_*str*	0.009393	No.14	No.10	-
*tf*_*compile*_*field*	0.009002	No.15	-	-
Node similarity (Γ)			9/15	9/15
Shared-Node Rate (Ψ)			13/15	

**Table 5:** Results of three algorithms performed on *Cflow*.

Fname	Noteworthiness	DNFM	PageRank	DegreeRank
*nexttoken*	0.139499	No.1	No.4	No.2
*get*_*token*	0.094022	No.2	-	-
*yylex*	0.094021	No.3	-	No.9
*tokpush*	0.094021	No.4	-	-
*hash*_*symbol*_*hasher*	0.072049	No.5	No.10	-
*hash*_*symbol*_*compare*	0.065406	No.6	No.11	-
*lookup*	0.063362	No.7	No.12	No.8
*putback*	0.043945	No.8	-	No.15
*ident*	0.037813	No.9	-	-
*get*_*symbol*	0.023505	No.10	-	-
*add*_*reference*	0.021972	No.11	-	-
*cleanup*_*stack*	0.021972	No.12	-	-
*reference*	0.017374	No.13	-	-
*expression*	0.014308	No.14	-	No.12
*mark*	0.014308	No.15	No.14	-
Node similarity (Γ)			5/15	5/15
Shared-Node Rate (Ψ)			9/15	

In Table 3, the experiment results show that there exist 8 functions which appeared in two top 15 function lists of *DNFM* and *PageRank* and 8 functions which appeared in two top 15 function lists of *DNFM* and *DegreeRank*, in which the *Share-node rate* for *DNFM* algorithm is equal to 10/15. As is shown in Table 4, for *Deadbeef*, there exist 9 functions which appeared in the experiment results of *DNFM* and *PageRank*, and 9 functions which appeared in the experiment results of *DNFM* and *DegreeRank*, in which the *Share-node rate* reaches 13/15. As the experiment result of *Cflow* is shown in Table 5, there exist 5 functions which appeared in experiment results of *DNFM* and *PageRank* and 5 functions which appeared in experiment results of *DNFM* and *DegreeRank*, and the *Shared-node rate* of *Cflow* equals 9/15. The *Node similarity* of each software is all lower than its *Shared-node rate*. All the above experiment data prove that *DNFM* algorithm based on software function call sequence has more advantages than the algorithms based on complex software network in finding noteworthy functions. Compared the functions obtained by *PageRank* with those by *DegreeRank* algorithm, it can be found that functions got by *DNFM* algorithm contain those which can be got by *PageRank* algorithm (such as, functions like *ngx*_*palloc*, *ngx*_*http*_*merge*_*locations* and *ngx*_*conf*_*handler* in *Ngnix*, *mutex*_*lock*, *mutex*_*unlock*, *conf*_*unlock* and *plt*_*unref* in *Deadbeef*, and *tokpush*, *get*_*symbol*, add_reference and expression in *Cflow*) but cannot be got by *DegreeRank* algorithm, or vice versa. Therefore, the method proposed in this paper, for it combines the advantages of those classic methods as *PageRank* algorithm and *DegreeRank* algorithm, is of higher efficiency and accuracy, and its results are more representative.

## Discussion and conclusions

In this paper, by taking functions as nodes and function calling as the order, we mapped software execution traces as function call sequences, and proposed a *DNFM* algorithm to find noteworthy functions in software sequences by analyzing those function call sequences. Firstly, we exploited an *Eliminate Repetitive patterns* algorithm to simplify initial function call sequences so as to reduce the influence from ring structure on mining noteworthy functions, and then generated patterns set from *SFS* by a designed pattern extraction algorithm. Secondly, after considering the frequencies and lengths of relevant *ESPs* and functions, we introduced two important evaluation indicators, because in the process of experiment, what the most important is to evaluate the impotence of each function. Thus we defined a new index *Noteworthiness* to measure the noteworthiness of functions. Finally, we compared *DNFM* with another two classic algorithms, namely, *PageRank* and *DegreeRank* algorithms which are conducted on complex network. The complex network weakly represents the software executions. It cannot reflect the sequence of function calls, which only describes whether there exists relationships between two functions. Comparing the results from these traditional algorithms mostly applied on complex network, the proposed model and method in this paper are much suitable for software analysis. The results also verify that the *DNFM* algorithm can identify noteworthy functions most effectively, and that the *shared-Node rate* of *DNFM* algorithm is highest in different software, even though the *Node similarity* between them is lower. For instance, the *Node similarity* of software *Cflow* is about 5/15 = 33.3%, but the *Shared-Node rate* achieves 9/15 = 60%. All of these prove that the *DNFM* algorithm can work on different software dynamic execution process and can identify noteworthy functions successfully, effectively and precisely. However, it should be noted that there is a disadvantage of the *DNFM* algorithm, that is, it will waste a lot of time in simplifying mega function call sequence, which needs to be solved in the near future.
